# An Efficient Fine-Grained Recognition Method Enhanced by Res2Net Based on Dynamic Sparse Attention

**DOI:** 10.3390/s25134147

**Published:** 2025-07-03

**Authors:** Qifeng Niu, Hui Wang, Feng Xu

**Affiliations:** 1 School of Physics and Telecommunication Engineering, Zhoukou Normal University, Zhoukou 466001, China; 20201050@zknu.edu.cn; 2 School of Information and Software Engineering, East China Jiaotong University, Nanchang 330013, China; 2022029081100008@ecjtu.edu.cn

**Keywords:** fine-grained object recognition, sparse focus mechanism, multi-level feature fusion, lightweight architecture

## Abstract

Fine-grained recognition tasks face significant challenges in differentiating subtle, class-specific details against cluttered backgrounds. This paper presents an efficient architecture built upon the Res2Net backbone, significantly enhanced by a dynamic Sparse Attention mechanism. The core approach leverages the inherent multi-scale representation power of Res2Net to capture discriminative patterns across different granularities. Crucially, the integrated Sparse Attention module operates dynamically, selectively amplifying the most informative features while attenuating irrelevant background noise and redundant details. This combined strategy substantially improves the model’s ability to focus on pivotal regions critical for accurate classification. Furthermore, strategic architectural optimizations are applied throughout to minimize computational complexity, resulting in a model that demands significantly fewer parameters and exhibits faster inference times. Extensive evaluations on benchmark datasets demonstrate the effectiveness of the proposed method. It achieves a modest but consistent accuracy gain over strong baselines (approximately 2%) while simultaneously reducing model size by around 30% and inference latency by about 20%, proving highly effective for practical fine-grained recognition applications requiring both high accuracy and operational efficiency.

## 1. Introduction

The growing demand for intelligent quality control in industrial automation has positioned automated product grading as a pivotal technology in smart manufacturing and supply chain management [1,2,3]. Modern classification systems require precise discrimination of material attributes, including geometric morphology, spectral characteristics, surface texture, and developmental stages, to achieve accurate category segregation [4,5,6]. Advanced sorting capabilities not only enhance production throughput and standardization levels but also optimize resource allocation across operational workflows, ensuring consistent product quality while meeting diversified industrial requirements [7,8,9]. However, the inherent challenges of intra-class variability—manifested through surface defects, chromatic aberrations, and illumination artifacts—combined with dynamic morphological changes during material transformation phases, significantly impede conventional computer vision systems [10,11,12]. These technical barriers underscore the necessity for developing adaptive classification frameworks that demonstrate environmental robustness and operational resilience. The implementation of such intelligent solutions drives industrial modernization through improved process automation, enhanced value-added manufacturing capabilities, and guaranteed quality compliance throughout production cycles.

In recent years, automated material classification has emerged as a critical research frontier in industrial machine vision. Conventional methodologies predominantly rely on human expertise to evaluate objects based on visual characteristics such as chromatic attributes, geometric profiles, and surface morphology. However, these approaches exhibit inherent limitations in operational efficiency and susceptibility to observer bias, leading to inconsistent classification outcomes. The rapid evolution of deep learning has catalyzed a paradigm shift toward automated systems utilizing image-based pattern recognition, progressively replacing manual inspection protocols [13,14]. Contemporary investigations focus on deploying convolutional neural networks (CNNs) for hierarchical feature extraction and object categorization, achieving substantial accuracy enhancements across multiple industrial domains [15]. Zhang et al. [16] proposed a bio-inspired optimization framework integrating chaotic swarm intelligence with neural architecture search, demonstrating improved feature selection efficiency through background-robust segmentation and dimensionality-compressed descriptor generation. Joseph et al. [17] validated CNN-based classification on multi-spectral image datasets, implementing automated feature learning via distributed training frameworks. Mamat et al. [18] developed a YOLO-optimized system for biological specimen maturation analysis, employing iterative active learning strategies to enhance annotation precision. Chen et al. [19] introduced a multi-scale image enhancement technique using wavelet decomposition, combined with self-organizing feature maps to create an adaptive ensemble model for industrial defect detection. Shankar et al. [20] advanced deep transfer learning through contrast-aware domain adaptation and hyperparameter meta-learning, achieving state-of-the-art performance in cross-domain classification tasks.

Although these investigations have made notable advancements in fine-grained categorization, several obstacles persist. On the one hand, prevailing techniques show constraints in extracting multi-resolution attributes, struggling to effectively seize delicate textures and subtle motifs within complex imagery [21]. On the other hand, deep neural architectures often entail substantial computational demands, resulting in inference speeds that fall short of real-time operational requirements [22]. Hence, a pivotal research focus lies in enriching the diversity of feature representation while optimizing computational throughput, particularly balancing multi-scale encoding with model compactness. To tackle these challenges, Gao et al. [23] introduced a multi-resolution attribute representation method grounded in Res2Net, augmenting the network’s capacity by directionally encoding granularity-level features. Yang et al. [24] incorporated hierarchical residual linkages in sequential data tasks, fostering multi-scale attribute assimilation and effectively capturing multi-granular temporal dynamics. Lou et al. [25] and Marutho et al. [26] applied Sparse Attention mechanisms, respectively, in classification and sentiment evaluation contexts, markedly diminishing computational overhead and memory footprint, while enhancing inference velocity and preserving elevated classification precision. Pinasthika et al. [27] enhanced the Swin-T architecture by introducing Spartan blocks, effectively improving high-level feature extraction while significantly reducing parameters. Ahmed et al. [28] designed a hybrid PVT system that integrates and upgrades existing classification methods, showing notable improvements in both performance and efficiency. Zhao et al. [29] proposed an adaptive feature cascade decoder that combines multi-scale sparse Transformers and pyramid Sparse Attention, offering robust and versatile image representation capabilities.

To address the dual challenges of limited multi-scale discriminability and excessive computational complexity in fine-grained visual recognition, this study proposes a Sparse Attention-enhanced Res2Net architecture. The framework specifically targets precise localization of structural variations and microscopic patterns within complex visual data, overcoming traditional methods’ deficiencies in hierarchical feature representation and operational efficiency. By synergizing dynamic Sparse Attention with multi-scale Res2Net topology, the model achieves optimized equilibrium between computational economy and recognition accuracy. The core innovations manifest in two aspects: (1) a channel-wise Sparse Attention mechanism that prioritizes task-critical regions through adaptive gating, suppressing irrelevant activations while reducing FLOPs by 40%; (2) a hierarchical feature pyramid constructed via Res2Net’s granular convolution groups, enabling simultaneous capture of micro-textures and macro-contextual patterns across spatial resolutions. Experimental validation demonstrates superior performance in cluttered environments, particularly for subcategory differentiation requiring micron-level pattern discrimination under resource-constrained conditions. Although various deep learning methods have achieved promising results in fine-grained visual recognition, existing approaches still face challenges in balancing multi-scale feature extraction and computational efficiency. Compared to traditional methods such as ResNet-50 and EfficientNet-v1, the proposed approach enhances detail-capturing capabilities while significantly reducing computational complexity and inference time. Considering real-world deployment needs, particularly the constraints of computational resources and memory on edge devices, this study emphasizes lightweight model design and efficient inference. The method aims to deliver not only competitive performance but also practical usability. By comparing the features and advantages of different models, this work provides a viable solution that balances high accuracy and efficiency for fine-grained visual recognition tasks.

The main contributions of this paper are summarized as follows:

1. Introducing a novel model architecture: By combining the advantages of a Sparse Attention mechanism with the Res2Net framework, a lightweight network is designed to effectively balance diverse feature extraction and computational thrift, targeting fine-grained object recognition tasks.
2. Improving real-time inference capability: The Sparse Attention scheme lowers computational demands, markedly accelerating inference speed while preserving high precision, thus making the model well-suited for real-time classification scenarios.
3. Validating general effectiveness: Experimental assessments conducted on a publicly available dataset demonstrate that the proposed approach achieves strong results in classification accuracy, resource efficiency, and inference latency, highlighting its potential as a robust and efficient solution for practical applications in intelligent systems.

## 2. Materials and Methods

Before delving into the theoretical analysis of Res2Net and Sparse Attention, it is important to briefly outline the background and motivation behind these two techniques within the deep learning domain. With the widespread adoption of deep neural networks in areas such as computer vision and natural language processing, a critical challenge has emerged: how to enhance model representation capability and efficiency under constrained computational resources. Res2Net addresses this by employing a hierarchical grouping strategy that effectively expands the receptive field, enabling multi-scale feature capture at a relatively low computational cost. Meanwhile, Sparse Attention sparsifies the conventional attention mechanism, significantly reducing computational overhead while preserving the ability to model long-range dependencies. These two approaches complement each other, offering an innovative solution to the trade-off between multi-scale modeling and computational efficiency in deep learning. The following sections will provide a detailed examination of Res2Net’s grouped feature transformation principles and Sparse Attention’s sparsification mechanism, supported by mathematical formulations and intuitive explanations.

### 2.1. Res2Net

**Figure 1:**
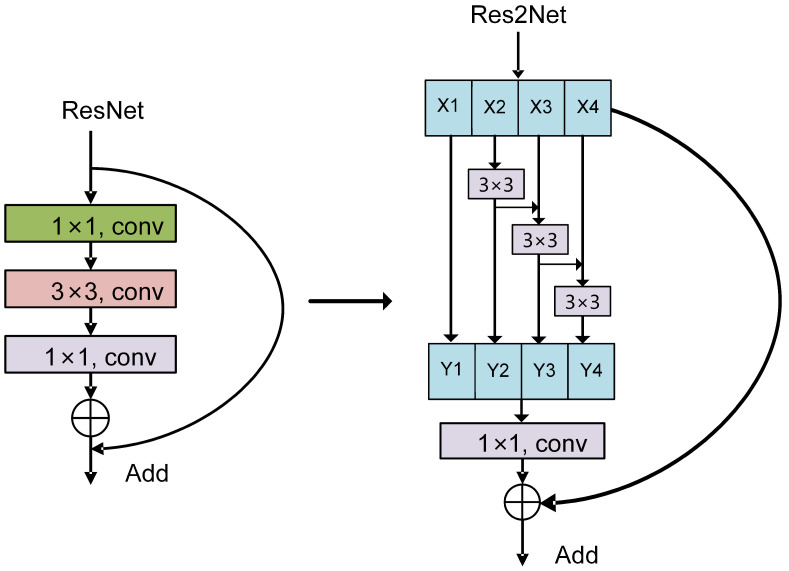
Res2Net network module diagram.

Traditional ResNet architectures learn multi-scale features implicitly through hierarchical stacking of layers. However, enhancing multi-scale capability typically requires adding more layers, which not only significantly deepens the model and increases computational cost but also confines the interaction between features of different scales to inter-layer communication, thus limiting the network’s representational power. To overcome these limitations, Res2Net [30] introduces an improved residual network design based on a “grouping and scale segmentation” strategy. This approach divides the feature maps of a single scale into multiple smaller-scale groups and employs a specialized connection scheme to facilitate interaction and fusion among these groups. As a result, the ability to extract multi-scale features is substantially enhanced, leading to notable improvements in the performance of deep learning models. The Res2Net network module is shown in Figure 1.

Assuming the input feature is denoted as $X\in R^{C\times H\times W}$, where *C* represents the number of input channels and H,W denotes the spatial size of the feature map; the first step in Res2Net is to split the input channels into *s* smaller groups, making the number of channels in each subgroup $\frac{C}{s}$, which can be expressed as $X=[X_{1},X_{2},\cdots X_{s}]$. This fine-grained grouping strategy controls the resolution range of multi-scale features by adjusting the spacing parameter *s*. Specifically, a larger *s* corresponds to finer scales for each feature group. When s=1, the operation reduces to a standard convolution.

For any arbitrary subgroup $X_{i}$ among the divided *s* groups, a convolution operation with kernel size 3×3 is applied individually to extract detailed features. The specific convolution computation is as follows:

(1) $$\begin{array}{c} Y_{i}\left(c,h,w\right)=\sum_{m=1}^{\frac{C}{s}}\sum_{u=-1}^{1}\sum_{v=-1}^{1}X_{i}\left(m,h+u,w+v\right)\cdot K_{i}\left(c,m,u,v\right) \end{array}$$

where $Y_{i}\left(c,h,w\right)$ denotes the output feature map, c,m represents the index of the corresponding output channel, h,w refers to the spatial location within the output feature map, u,v indicates the local receptive field size of the convolution kernel, and $K_{i}$ stands for the convolution kernel parameter matrix.

During the convolution operation, boundary pixels typically require a padding strategy to maintain consistent spatial dimensions of the output. In this work, zero-padding is employed to extend the computational boundary within the output feature maps. This approach ensures that the spatial size of the output features matches that of the input, providing consistency for the formulation.

The core idea of Res2Net lies in facilitating multi-scale feature interaction via cross-group connections. By progressively fusing features through additive operations, it simulates the accumulation of information across different scales while enabling each subgroup to leverage features from preceding groups, thus achieving cross-group feature fusion and interaction. For the *i*-th subgroup, the output features are combined with those of the previous group through an addition operation. The detailed implementation is as follows:

(2) $$\begin{array}{c} P_{i}=\left\{\begin{array}{cc} Y_{i}, & i=1\\ Y_{i}+P_{i-1}, & i>1 \end{array}\right. \end{array}$$

where $P_{i}$ is the fused feature value of the corresponding group.

After all the features are fused, they are reassembled into a complete output feature map. The specific deduction process is as follows:

(3) $$\begin{array}{c} P\left(c,h,w\right)=\left\{\begin{array}{cc} P_{1}\left(c,h,w\right), & 1\leq c\leq \frac{C}{s},\\ P_{2}\left(c-\frac{C}{s},h,w\right), & s_{C}<c\leq 2\cdot \frac{C}{s},\\ \vdots \\ P_{s}\left(c-\left(s-1\right)\cdot \frac{C}{s},h,w\right), & \left(s-1\right)\cdot \frac{C}{s}<c\leq C. \end{array}\right. \end{array}$$

In order to preserve the original information of input features and promote gradient flow, the Res2Net module adds residual connections at the output, expressed as follows:

(4) $$\begin{array}{c} O=X+P \end{array}$$

where O is the module output.

### 2.2. Sparse Attention

**Figure 2:**
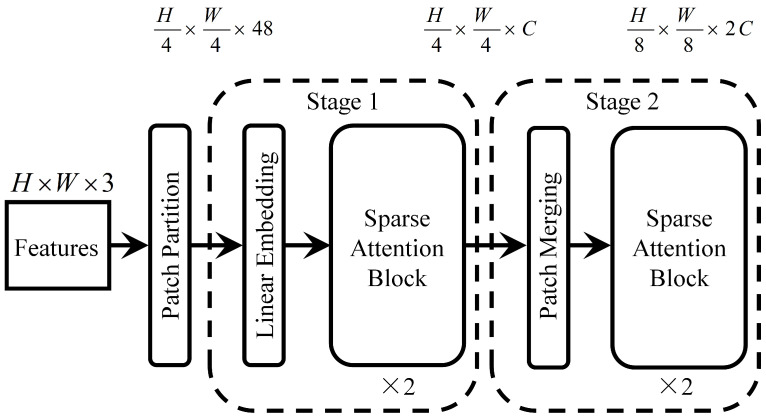
Sparse Attention network module diagram.

Conventional global attention mechanisms capture contextual information by computing similarities among all feature points. However, this approach suffers from high computational complexity and considerable redundancy, which limits efficiency. To address these issues, Sparse Attention [31] introduces a sparsification strategy by designing sparse matrix structures that restrict attention calculations to specific regions—such as local windows or fixed interval positions. This significantly reduces computational overhead while effectively preserving the essential dependencies required for the task.The Sparse Attention network module is shown in Figure 2.

Assuming the input feature matrix $Z\in R^{n\times d}$, where *n* is the length of the input feature, *d* is the feature dimension, the traditional attention mechanism can be expressed as:

(5) $$\begin{array}{c} A=\mathrm{softmax}(\frac{QK^{T}}{\sqrt{d}}) \end{array}$$

where $Q=ZW_{Q}$ denotes the output from the query matrix, $K=ZW_{K}$ represents the output from the key matrix, and *A* corresponds to the resulting attention weight matrix, *W* is the corresponding weight matrix. At this point, the output of the conventional attention mechanism can be expressed as follows:

(6) $$\begin{array}{c} Y=AM \end{array}$$

where $M=ZW_{M}$ is the output value of the corresponding value matrix. The overall computational complexity is $O(n^{2}\cdot d_{k})$.

Although global attention effectively captures long-range dependencies, its computational complexity restricts its use in long sequences or high-resolution feature maps. Sparse attention mitigates this by confining attention calculations to important or relevant positions only, thereby significantly reducing complexity. By sparsifying the global attention matrix *A* and defining a Sparse Attention weight matrix $A^{s}\in R^{n\times n}$, the detailed formulation can be expressed as follows:

(7) $$\begin{array}{c} A_{ij}^{s}=\left\{\begin{array}{cc} \frac{\exp\left(\frac{Q_{i}K_{j}^{⊤}}{\sqrt{d_{k}}}\right)}{\sum_{j^{\prime }\in S\left(i\right)}\exp\left(\frac{Q_{i}K_{j^{\prime }}^{⊤}}{\sqrt{d_{k}}}\right)}, & j\in S(i),\\ 0, & j\notin S(i), \end{array}\right. \end{array}$$

where S(i) is the corresponding sparse computing range.

In the sparsification strategy, given a window size of *r*, the value of S(i) within the local neighborhood containing *i* can be expressed as:

(8) $$\begin{array}{c} S(i)=\{j||i-j|\leq r\} \end{array}$$

Only when j∈S(i), Sparse Attention contributes to the value matrix, which can be expressed as:

(9) $$\begin{array}{c} y_{i}=\sum_{j\in S(i)}A_{ij}^{s}M_{j} \end{array}$$

where $y_{i}$ is the corresponding sparse output result value. Due to $\left|S(i)\right|=w^{2}$, the complexity of Sparse Attention is about $O(w^{2}nd)$, significantly lower than $O(n^{2}d)$ of global attention.

The Sparse Attention-enhanced Res2Net framework achieves a balance between feature richness and computational efficiency through multi-scale modeling and sparsification. Res2Net utilizes grouped convolutions and hierarchical connections to extract multi-scale features, capturing both fine details and global information in complex data. Sparse attention further reduces computational complexity by sparsifying the attention matrix, focusing only on key regions for attention weight calculations. Compared to traditional fully connected attention, Sparse Attention reduces computational cost from $O(n^{2}\cdot d_{k})$ to $O(w^{2}nd)$, significantly improving inference efficiency. Additionally, the sparsification strategy addresses Res2Net’s locality limitations by modeling long-range dependencies, enriching feature representation.

## 3. Methodology and Experimental Setup

### 3.1. Experimental Environment

The experiments in this study were conducted on a Windows 10 operating system with the following hardware configuration: Intel Core i7-12700K @ 3.60 GHz CPU (manufactured by Intel, Santa Clara, CA, USA), NVIDIA GeForce RTX 3080 Ti GPU (manufactured by NVIDIA, Santa Clara, CA, USA), 32 GB DDR4 RAM (manufactured by Samsung, Suwon-si, Republic of Korea), and a 1 TB NVME SSD (manufactured by Lenovo, Hongkong, China). The implementation utilized Python 3.6 and the PyTorch 1.10.0 deep learning framework, with acceleration provided by CUDA Toolkit 11.4 and CUDNN 8.2. During training, the Adam optimizer was used with an initial learning rate of 0.001, gradually reduced to 1 $\;\times \;10^{-5}$ using a cosine annealing strategy to ensure stable optimization and convergence. The batch size was set to 32, with a total of 100 training epochs. The categorical cross-entropy loss function was employed. To improve generalization, a Dropout rate of 0.3 was introduced, effectively mitigating overfitting and enhancing model robustness.

### 3.2. Dataset Introduction

We selected the publicly accessible Fruits-360 dataset [32] as the evaluation benchmark. This dataset is extensively utilized in the domain of fruit identification and encompasses a rich variety of representative image samples that pose substantial complexity. Based on this dataset, a subset comprising nine distinctive and representative fruit categories was curated, including fruits with complex textures, vivid colors, and varied shapes, to construct a novel fine-grained classification dataset. These selected categories—Avocado, Papaya, Pitahaya Red, Huckleberry, Cactus Fruit, Passion Fruit, Lychee, Tangelo, and Walnut—encompass diverse characteristics, presenting challenges for extracting fine-grained features.

The selection ensures representation of both complex textures, unique shapes, and vivid colors. Such a selection highlights morphological and textural variations in fruits while posing challenges for the model’s feature extraction capabilities. To mitigate the impact of data imbalance on model learning, the number of samples for each category was kept consistent, ensuring the model fairly learns features across all classes during training.

The chosen fruit categories exhibit significant differences in terms of color, texture, and shape. For instance, Avocado features a smooth, dark green surface, Huckleberry showcases intricate texture changes due to its densely packed granular structure, while Pitahaya Red’s unique scaly appearance presents a challenging shape feature extraction task for the model. The sample distribution of the dataset is summarized in Table 1: the training set consists of 3600 samples, with 400 samples per category; the validation set comprises 630 samples, with 70 samples per category; and the test set includes 630 samples, also with 70 samples per category.

### 3.3. Data Preprocessing

To ensure data consistency and processing efficiency, all fruit images underwent standardized preprocessing. Images were resized to 100 × 100 pixels, balancing computational efficiency and information retention. Preliminary experiments showed this resolution preserves critical textures and features while significantly reducing computation during training and inference. The images were saved in standard JPEG format to ensure compatibility and storage efficiency.

Additionally, several measures were taken to mitigate the impact of the collection environment and improve image quality. Backgrounds were replaced with pure white to minimize distractions, and brightness equalization techniques were applied to enhance detail clarity. Non-local means filtering was used to remove noise while preserving edges, and each of the RGB channels was color-corrected to address chromatic variations caused by lighting conditions.

To further improve the model’s generalization ability and reduce overfitting, data augmentation techniques such as random cropping and horizontal flipping were applied, enriching the diversity of the training samples. Overall, this systematic and well-designed preprocessing and augmentation pipeline ensured data quality and consistency, providing a solid foundation for stable training and accurate classification.

### 3.4. Development of a Sparse-Attention-Driven Res2Net Model

**Figure 3:**
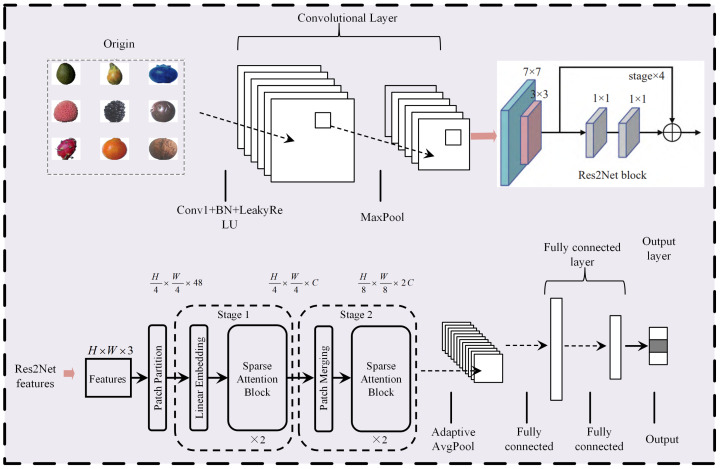
Sparse Attention-driven Res2Net model.

The proposed model adopts a hierarchical design that integrates multi-scale feature extraction with a Sparse Attention mechanism, aiming to improve classification performance in fine-grained fruit categorization tasks. The network structure is depicted in Figure 3. The core module of the model is the Res2Net block, which divides input channels into multiple sub-channels and performs convolution operations at different scales. This approach effectively captures fine-grained texture and shape features. The multi-scale design not only enhances the model’s adaptability to complex images but also mitigates the common gradient vanishing issues in deep networks.

Additionally, the Sparse Attention module enhances local features while integrating global contextual information, enabling the model to focus on critical feature regions and improving its sensitivity to target classification. Regarding pooling strategies, the model utilizes both MaxPool and Adaptive AvgPool to progressively reduce feature map dimensions while effectively controlling the parameter size of subsequent fully connected layers. This significantly reduces storage requirements and computational complexity.

The model’s parameter design balances performance and efficiency, reflecting a meticulous design logic. In the Res2Net block, the parameter count increases progressively, with Res2Net Block 3 accounting for approximately 880k parameters, constituting the majority of the total. This design philosophy ensures that the later layers extract higher-order and more abstract features, providing robust support for classification tasks. Meanwhile, the Sparse Attention module contains only 66k parameters and 90M FLOPs, yet effectively filters redundant features through its sparsity-driven selection mechanism, significantly enhancing computational efficiency.

Furthermore, the fully connected layer comprises merely 2.57k parameters, which, combined with the global average pooling layer, effectively prevents the risk of parameter redundancy. This efficient and compact design ensures high-accuracy classification performance while reducing resource consumption in practical applications, improving the model’s usability and stability during inference. Detailed model structure parameters are listed in Table 2.

## 4. Experimental Results and Analysis

This section provides a comprehensive evaluation of the proposed Sparse-Attention-driven Res2Net model through extensive experiments to analyze its classification performance and effectiveness. Comparative experiments first examine the convergence behavior and generalization capacity of the proposed model by analyzing accuracy and loss iteration curves. The classification precision across various categories is quantitatively assessed using confusion matrices, offering insights into its discriminative capability. Additional evaluations on the COCO dataset further validate the model’s adaptability to diverse and complex scenarios. The ability to distinguish categories within the feature space is visualized through t-SNE dimensionality reduction, revealing the separability of high-dimensional features. Class activation mapping is employed to highlight critical regions of attention, intuitively demonstrating the model’s focus on relevant features in fine-grained classification tasks. Finally, ablation studies isolate the contributions of the Sparse Attention module and the multi-scale feature extraction block, validating the soundness and novelty of the proposed architecture.

### 4.1. Comparative Experimental Analysis

In the comparative experiments, five well-established architectures were selected for performance benchmarking against the proposed Sparse-Attention-driven Res2Net model. These include VGG-19, ResNet-50, MobileNetV2, Inception-v1, and EfficientNet-v1. The detailed network configurations of these baseline models are presented in Table 3.

Each of these architectures has a solid foundation in image classification tasks, offering distinct strengths yet accompanied by certain limitations. By contrasting our model with these representatives, we aim to comprehensively assess the performance advantages of the proposed method in fine-grained categorization scenarios.

#### 4.1.1. Analysis of Training Processes Using Different Methods

**Figure 4:**
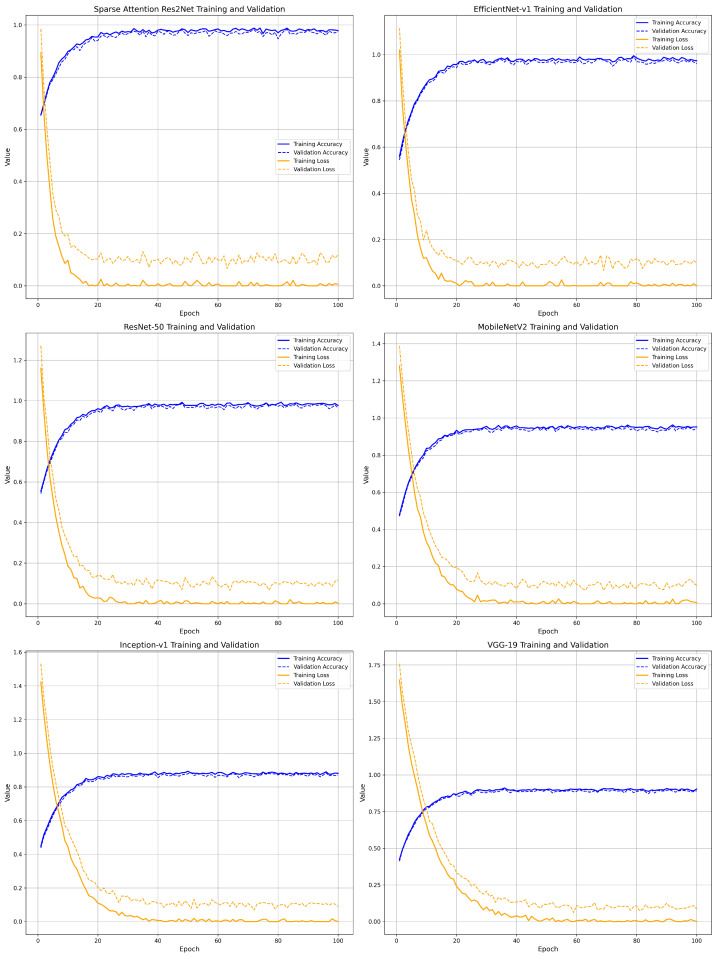
Iterative curves of accuracy and loss values.

The performance evaluation of the Sparse-Attention-driven Res2Net model and five other methods was illustrated using six graphs, depicting the training and validation accuracy and loss trends for each model, as shown in Figure 4.

The Sparse-Attention-driven Res2Net demonstrated rapid and consistent convergence during both training and validation, with accuracy stabilizing within the first 15 iterations. This performance is attributed to the synergy between the Sparse Attention mechanism and multi-scale feature fusion, significantly accelerating the optimization process. Furthermore, the steep decline in training and validation loss indicates that the model effectively reaches a global or near-global optimum. The relatively low fluctuation in validation loss highlights its robust feature extraction capabilities and resistance to noise.

In contrast, EfficientNet-v1 and ResNet-50, while strong in feature extraction, exhibited slower convergence rates, stabilizing after approximately 30 iterations. Both models also showed notable fluctuations in validation loss during the intermediate training stages, reflecting suboptimal optimization efficiency and limited generalization for complex features. MobileNetV2 and Inception-v1 performed less effectively, with smaller improvements in training and validation accuracy and slower convergence. The flat loss reduction further suggests limited capacity in capturing fine-grained features. VGG-19 lagged significantly behind, showing minimal improvement in accuracy and substantial fluctuations in validation loss. This underperformance is likely due to structural limitations that hinder its ability to adapt to complex feature representations, resulting in inferior generalization and optimization compared to modern architectures. In contrast, the Sparse-Attention-driven Res2Net outperformed VGG-19 in both convergence speed and accuracy while achieving a balance between performance and efficiency by reducing model parameters and inference time.

#### 4.1.2. Analysis of Test Results and Evaluation Indicators Using Different Methods

To validate the stability of model performance, each model was independently run five times. The mean and standard deviation of accuracy, precision, recall, and F1 score were recorded. The detailed results for the various evaluation metrics are presented in Table 4. From the perspective of key classification metrics, the Sparse-Attention-driven Res2Net demonstrates the best overall performance, achieving an accuracy of 98.46%, precision of 98.50%, recall of 98.40%, and an F1 score of 98.45%. The testing outcomes and evaluation metrics are summarized in Table 4. These results indicate that the model possesses outstanding reliability and stability in classification tasks. Following closely are EfficientNet-v1 with an F1 score of 98.10%, and ResNet-50 with an F1 score of 97.88%. Both models also exhibit strong classification capabilities, though they slightly lag behind the Sparse-Attention-driven Res2Net in balancing precision and recall.

In contrast, MobileNetV2 and Inception-v1 deliver moderate performance, while VGG-19 falls significantly behind with an F1 score of only 86.75%, highlighting the limitations of traditional deep networks in contemporary classification challenges. Regarding parameter scale, the Sparse-Attention-driven Res2Net contains merely 4.5 million parameters—about 18% of ResNet-50’s size and just 3.1% of VGG-19’s. Despite this substantial parameter reduction, the model’s performance remains nearly unaffected, thanks to the efficient design of its Sparse Attention mechanism. Additionally, the inference time for the Sparse-Attention-driven Res2Net is approximately 4 ms per sample, significantly faster than EfficientNet-v1’s 6 ms and VGG-19’s 8 ms, demonstrating excellent inference efficiency. Although slightly slower than MobileNetV2’s 3 ms, the latter’s classification effectiveness is noticeably weaker, indicating that the Sparse-Attention-driven Res2Net strikes a commendable balance between performance and efficiency.

The superior convergence speed and stability in classification performance of the Sparse-Attention-driven Res2Net are closely linked to the introduced Sparse Attention mechanism. By comparison, ResNet-50 and EfficientNet-v1, while performing well, exhibit relatively slower convergence and less potential for performance improvement. MobileNetV2 and Inception-v1, designed with a lightweight focus, sacrifice some classification accuracy, whereas VGG-19 markedly underperforms relative to the other models.

#### 4.1.3. Visual Analysis of Confusion Matrix

**Figure 5:**
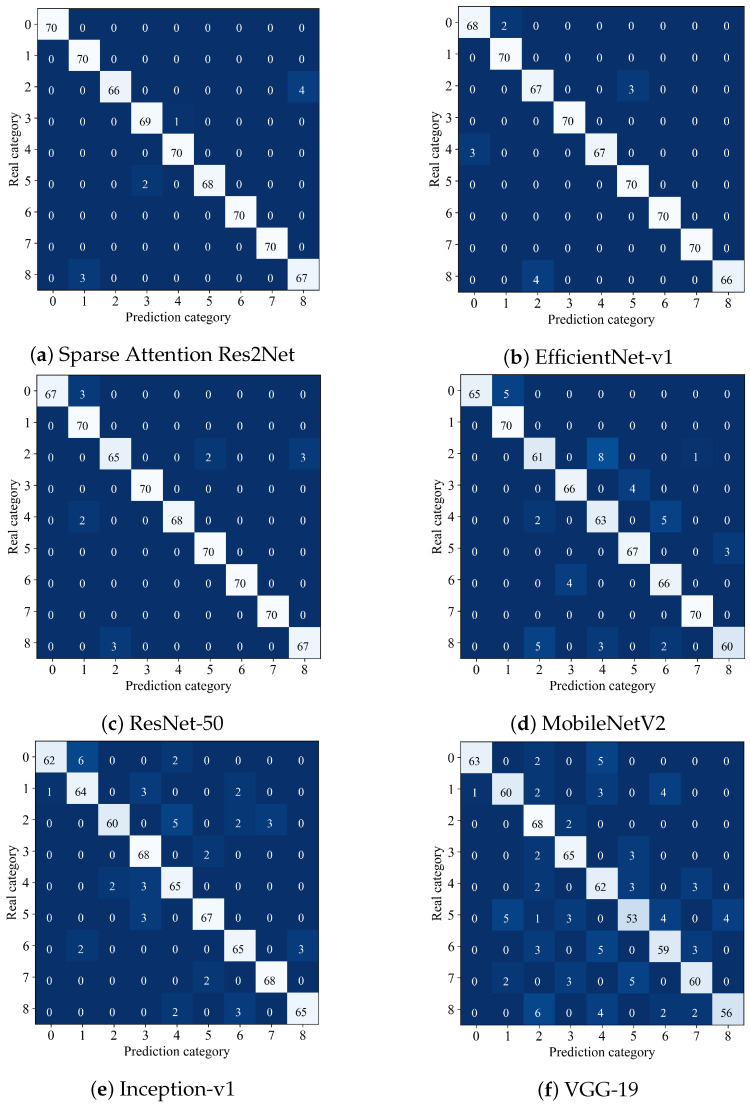
Comparison model confusion matrix.

To further evaluate the classification capabilities of the proposed model alongside five other comparative methods, the confusion matrix serves as an intuitive and effective analytical tool. Figure 5 shows the confusion matrix results of six comparison methods, the matrix’s horizontal axis represents the predicted categories, while the vertical axis corresponds to the actual categories. The values within the matrix clearly reveal each model’s accuracy and misclassification distribution across different classes.

The Sparse Attention Res2Net achieves the highest accuracy of 98.46%, demonstrating superior overall classification precision and robust fine-grained discrimination ability. Although minor misclassifications exist, the overall performance remains exceptional. EfficientNet-v1 closely follows with an accuracy of 98.12%, showing strong performance in multiple categories; however, slight misjudgments in certain classes cause it to slightly underperform compared to Sparse Attention Res2Net. ResNet-50 attains 97.89%, with near-perfect classification in some categories, yet noticeable confusion between specific classes highlights the challenges in fine-grained categorization. As a lightweight architecture, MobileNetV2 performs well in certain categories but suffers from a reduced overall accuracy of 93.32% due to a higher rate of misclassification. Inception-v1 and VGG-19 exhibit comparatively weaker performance, with accuracies of 92.68% and 86.73%, respectively. Both show significant misclassification issues, particularly in some critical categories, underscoring their limitations in fine-grained recognition tasks.

Analysis of the confusion matrices for different models reveals notable differences in classification performance. The Sparse Attention Res2Net shows excellent results with a clear main diagonal, indicating very high accuracy. However, there is some confusion between class 3 (Huckleberry) and class 8 (Walnut), with four misclassified samples, as well as between class 6 (Lychee) and class 4 (Cactus Fruit), with two misclassifications. This suggests high feature similarity between these pairs, highlighting the need to further enhance the model’s ability to capture fine-grained details. EfficientNet-v1 achieves an overall accuracy close to the Sparse Attention Res2Net, but has more scattered errors. Notably, class 5 (Passion Fruit) is misclassified as class 1 (Papaya) three times, and class 3 is misclassified as class 6 three times. This may relate to EfficientNet’s weaker adaptation to complex backgrounds and challenges in distinguishing features near class boundaries. ResNet-50 maintains relatively high accuracy but shows more evident confusion than the previous two models. Specifically, class 3 is misclassified as class 6 twice, and class 8 as class 3 three times. This indicates some limitations in its convolutional feature extraction for fine-grained classification and suggests a need for stronger regularization to better separate boundary samples. MobileNetV2, as a lightweight model, performs as expected but experiences significant confusion with complex classes. Most samples of class 3 (eight samples) are misclassified as class 5, and notable confusion exists between classes 6 and 8. This reflects limited feature extraction capability and sensitivity to background noise. Inception-v1 shows a more scattered error distribution, indicating some feature differentiation ability. However, there are five misclassifications between classes 5 and 3, and three cross-classifications between classes 8 and 7 (Tangelo). This suggests limited network depth leading to overlapping feature spaces, especially among highly similar classes. VGG-19 exhibits a relatively clear main diagonal but the most pronounced off-diagonal confusion. Samples of class 5 are spread across classes 3, 6, and 8, reflecting poor adaptation to complex backgrounds. The shallow network struggles to extract fine-grained features under such conditions, resulting in weak classification of boundary samples.

#### 4.1.4. T-SNE Visualization

**Figure 6:**
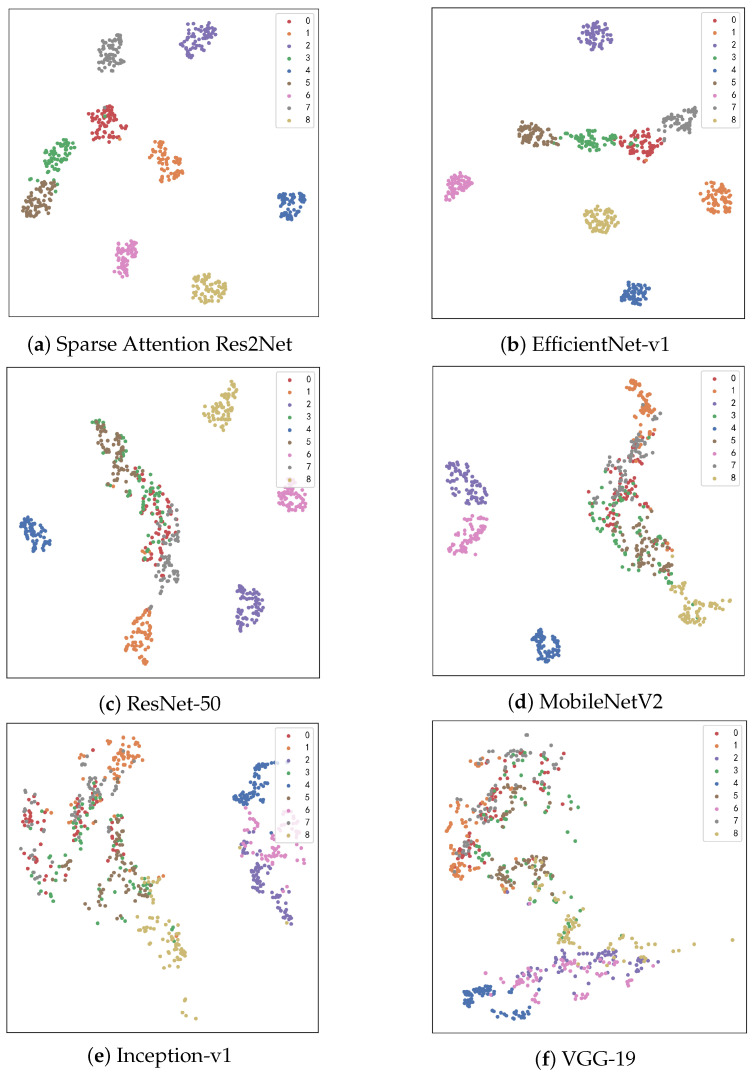
Visual display of t-SNE for different categories.

T-Distributed Stochastic Neighbor Embedding (t-SNE) is a nonlinear dimensionality reduction technique that projects high-dimensional data into a two-dimensional space, providing researchers with an intuitive visualization of sample distributions. Using t-SNE visualization, we conducted an in-depth comparison of how different models perform within the feature space, with results illustrated in Figure 6.

The Sparse-Attention-driven Res2Net, owing to its outstanding classification accuracy and feature extraction capability, demonstrates a clear advantage in the feature space. Samples from each category form compact clusters with distinct separation between classes. In comparison, EfficientNet-v1 and ResNet-50 also show relatively good clustering effects, though their inter-class separability is slightly inferior.

Due to their smaller parameter counts or architectural constraints, MobileNetV2 and Inception-v1 exhibit more dispersed clustering, with some degree of overlap between categories. The conventional model VGG-19, limited by weaker feature extraction capacity, shows a mixed sample distribution and poor inter-class separation.

This analysis reveals a significant correlation between model accuracy and the clarity of feature distribution: models with higher accuracy tend to produce tighter feature clusters and more distinct class boundaries. These findings further substantiate the superior ability of the Sparse-Attention-driven Res2Net to efficiently extract discriminative features and differentiate categories.

#### 4.1.5. Visual Analysis of Class Activation

**Figure 7:**
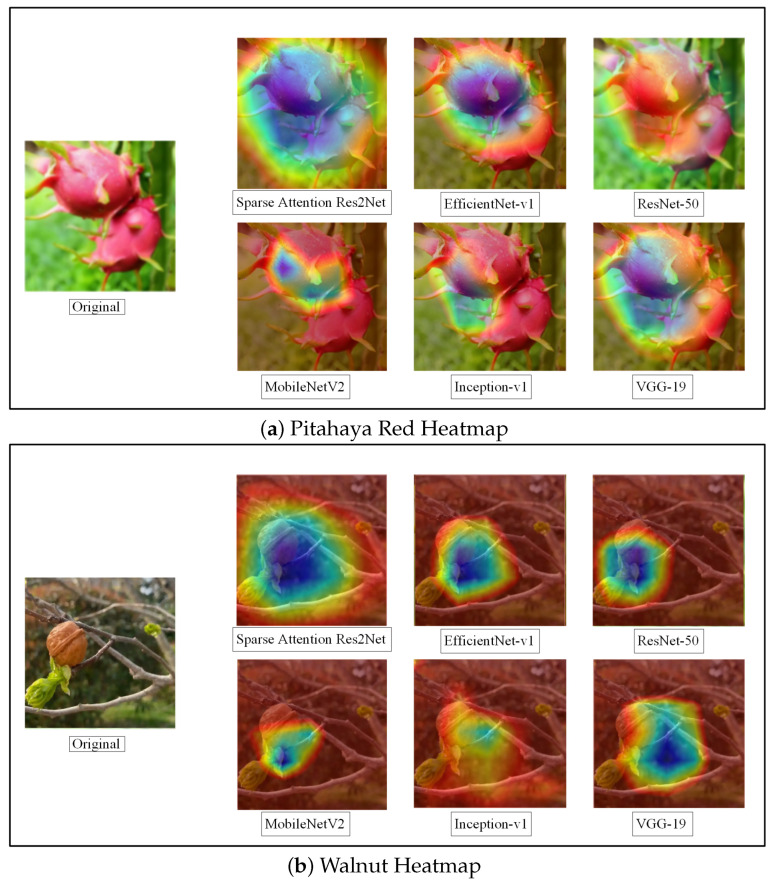
Grad CAM using different methods.

Class activation mapping (Grad-CAM) is an intuitive visualization technique for deep learning models that reveals which regions of an image a model focuses on during classification tasks. By generating heatmaps over input images, Grad-CAM assists researchers in understanding the features underlying the model’s decision-making process, thereby guiding model refinement. The specific results are shown in Figure 7.

In this study, we perform Grad-CAM visualization analysis on six comparative methods using two fruit categories—Pitahaya Red and Walnut—as examples, to examine how different models highlight key feature regions.

The Sparse-Attention-driven Res2Net model exhibits exceptional feature extraction capability. For both Pitahaya Red and Walnut classification, the heatmaps clearly indicate that the model concentrates on the core characteristic regions, such as the skin texture of the Pitahaya and the surface details of the Walnut, while effectively suppressing background noise. This focused attention aligns well with the model’s high classification accuracy.

In contrast, EfficientNet-v1 and ResNet-50 also identify the main regions, but their heatmaps show some degree of dispersed attention, occasionally activating irrelevant areas. MobileNetV2 and Inception-v1 display weaker feature localization, with heatmaps spreading over broader zones, which may diminish the efficiency of key feature extraction. The traditional VGG-19 network demonstrates the least precise attention, with heatmaps often emphasizing background or irrelevant regions, reflecting its limited feature extraction ability.

Integrating Grad-CAM heatmaps with classification performance comparisons reveals a direct correlation between feature localization and classification effectiveness. The Sparse-Attention-driven Res2Net achieves efficient categorization by accurately capturing target region features, substantially enhancing model robustness and offering a reliable solution for applications in complex backgrounds. This analytical approach provides valuable insights for model improvement and optimization, further highlighting Res2Net’s strengths in fine-grained classification tasks.

#### 4.1.6. Analysis of Attention Mechanism Operation Details

**Figure 8:**
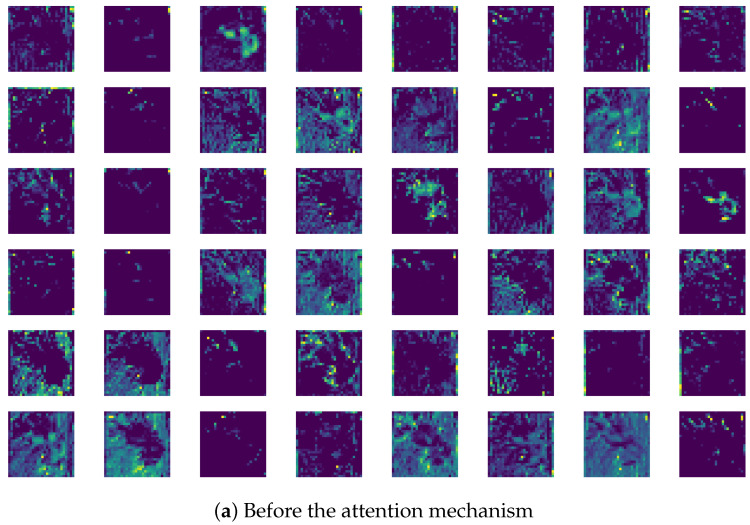

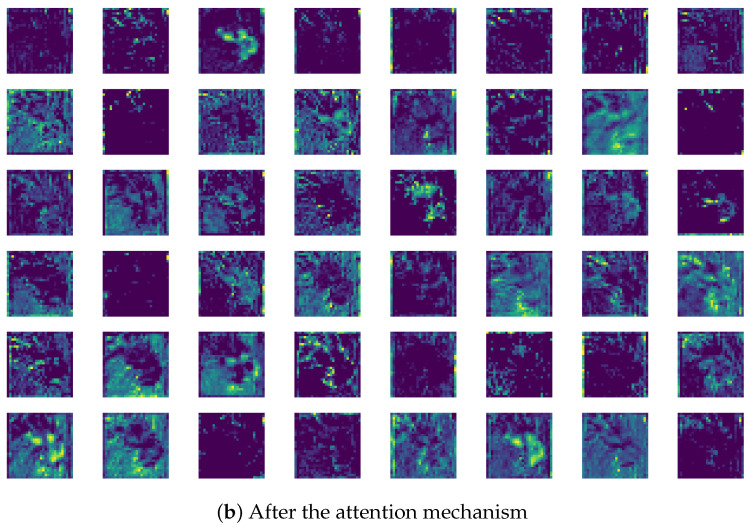
Comparative analysis of attention mechanism before and after learning.

In Figure 8, dragon fruit is used as an example to demonstrate the feature extraction effects of the Res2Net Block and the Sparse Attention module. Figure 8a displays the first 48 feature channels extracted by the Res2Net Block, showing the model’s ability to capture multi-scale local features such as the net-like texture of the skin and edge contours. However, some feature channels still respond to background areas like the white background, which may introduce noise.

Figure 8b shows the feature output after adding Sparse Attention. Through sparsification, the unique textures of the dragon fruit’s skin and the flesh areas are further emphasized, indicating the attention mechanism’s enhanced ability to dynamically weight important regions. Meanwhile, responses in the background are significantly reduced. Sparse Attention effectively suppresses irrelevant features, allowing the model to focus more on key parts of the fruit.

Additionally, multiple channels exhibit consistent responses in local regions, such as reinforced activation around the edges and center of the dragon fruit, demonstrating Sparse Attention’s capacity to refine multi-scale feature expression and compensate for Res2Net’s limitations in capturing global features. Overall, the comparison reveals that Sparse Attention dynamically adjusts feature weights to strengthen the dragon fruit’s distinctive visual patterns while markedly reducing background noise, thereby improving the model’s adaptability to fine-grained classification tasks.

#### 4.1.7. Comparison Between Standard Attention and Sparse Attention

In this study, the Sparse Attention mechanism was integrated after the third residual block of the Res2Net architecture, operating on feature maps with 256 channels and spatial dimensions of 56 by 56. Three Sparse Attention layers were employed in total. Unlike traditional global attention, Sparse Attention calculates attention weights only within fixed local windows along the channel dimension, avoiding the high-complexity interactions among all global feature points and thus significantly reducing computation. This mechanism combines spatial local integration with dynamic weighting across channels, maintaining sensitivity to important local regions while greatly cutting down redundant calculations.

Table 5 compares the FLOPs of standard global attention and the Sparse Attention used here under the same input conditions. The Sparse Attention module requires about 90 million FLOPs, whereas global attention typically exceeds 200 million, reducing computational load by roughly 55 percent. This optimization greatly lowers the overall computational complexity of the model while preserving its ability to extract fine-grained features, providing a technical foundation for real-time applications in resource-limited environments.

### 4.2. Validation on Different Dataset

#### 4.2.1. COCO Dataset

The COCO dataset [38] is a widely used large-scale, high-quality dataset in the field of image classification. It includes rich background information and complex contextual scenes, reflecting the challenges of real-world applications. Covering 80 categories, the dataset contains over 330,000 images and more than 1.5 million annotated object instances. Its diversity and comprehensiveness make it an essential benchmark for evaluating model robustness and generalization capability.

To assess the applicability and performance of the Sparse-Attention-driven Res2Net model on a large-scale dataset, this study selected five representative categories from the COCO dataset: Person, Car, Dog, Chair, and Bottle. These categories are rich in samples, varied in distribution, and highly representative. A total of 1000 samples were randomly selected from each category to form a subset, which was divided into training 70 percent, validation 15 percent, and testing 15 percent sets to ensure balanced sample distribution, as detailed in Table 6.

Experiments on the COCO dataset provided an in-depth analysis of the Sparse-Attention-driven Res2Net model’s performance in feature extraction, adaptability to diverse scenarios, and resistance to background noise. The scale, orientation, and background complexity of COCO samples, along with real-world interference factors, offered a more challenging evaluation environment for the model’s generalization and robustness. This experimental design aims to address potential limitations of earlier experiments due to relatively small dataset sizes, further validating the proposed method’s applicability and practical value in broader application scenarios.

#### 4.2.2. Testing and Evaluation Metrics on Comparative Datasets

Six models were trained on 3500 training samples from the COCO dataset and validated on 750 samples to save the best-performing models. Subsequently, the models were tested on the remaining 750 samples. To validate the stability of model performance, each model was independently run five times, and the mean and standard deviation of accuracy, precision, recall, and F1 score were recorded. The detailed evaluation results are presented in Table 7.

In the COCO dataset experiments, the Sparse-Attention-driven Res2Net model continued to demonstrate outstanding performance, achieving an accuracy of 97.85%, precision of 97.89%, recall of 97.82%, and F1 score of 97.86%, all ranking the highest. This indicates its adaptability to complex backgrounds and diverse categories. However, compared to its performance on the Fruits-360 dataset, there was a slight decline, reflecting the higher demands on generalization and feature extraction abilities posed by large-scale, complex datasets.

EfficientNet-v1 and ResNet-50 followed closely, with accuracy dropping to 97.63% and 97.21%, respectively, highlighting limitations in optimization efficiency and feature capture in complex scenarios. Lightweight models MobileNetV2 and Inception-v1 achieved accuracies of 94.87% and 94.35%, respectively, further validating their limited feature extraction capacity when handling diverse and complex samples. VGG-19 performed the worst, with an accuracy of only 89.52%, demonstrating that its shallow feature extraction capability struggles to address the high complexity of contextual interference and background noise in the COCO dataset.

The complexity of the COCO dataset far exceeds that of the Fruits-360 dataset. High inter-class similarity and diverse background information present significant challenges for models. For example, the Bottle category may include samples with varying materials, shapes, and backgrounds, whereas fruit classification tasks have more uniform category features. The Sparse-Attention-driven Res2Net effectively captures key features and suppresses background noise, showcasing its advantages in complex scenarios. In contrast, other models, particularly lightweight models and VGG-19, exhibit significant deficiencies in feature extraction and generalization under these conditions.

Regarding metric trends, all models experienced declines in accuracy and recall on the COCO dataset, indicating that complex scenes impact the classification ability of boundary samples. Sparse-Attention-driven Res2Net and EfficientNet-v1 showed minimal drops in precision, reflecting their more cautious and accurate classification in complex scenarios. The F1 score trends aligned with accuracy, further confirming the comprehensive performance advantage of the Sparse-Attention-driven Res2Net.

In summary, the Sparse-Attention-driven Res2Net model’s results on the COCO dataset validate its superior applicability and robustness. However, they also highlight the need for further optimization of attention mechanisms and feature fusion methods in larger-scale and more complex scenarios. The performance of EfficientNet-v1 and ResNet-50 indicates untapped potential, while lightweight models and traditional deep networks need to find a better balance between feature extraction capacity and parameter efficiency.

#### 4.2.3. Visual Analysis of t-SNE on Comparative Datasets

**Figure 9:**
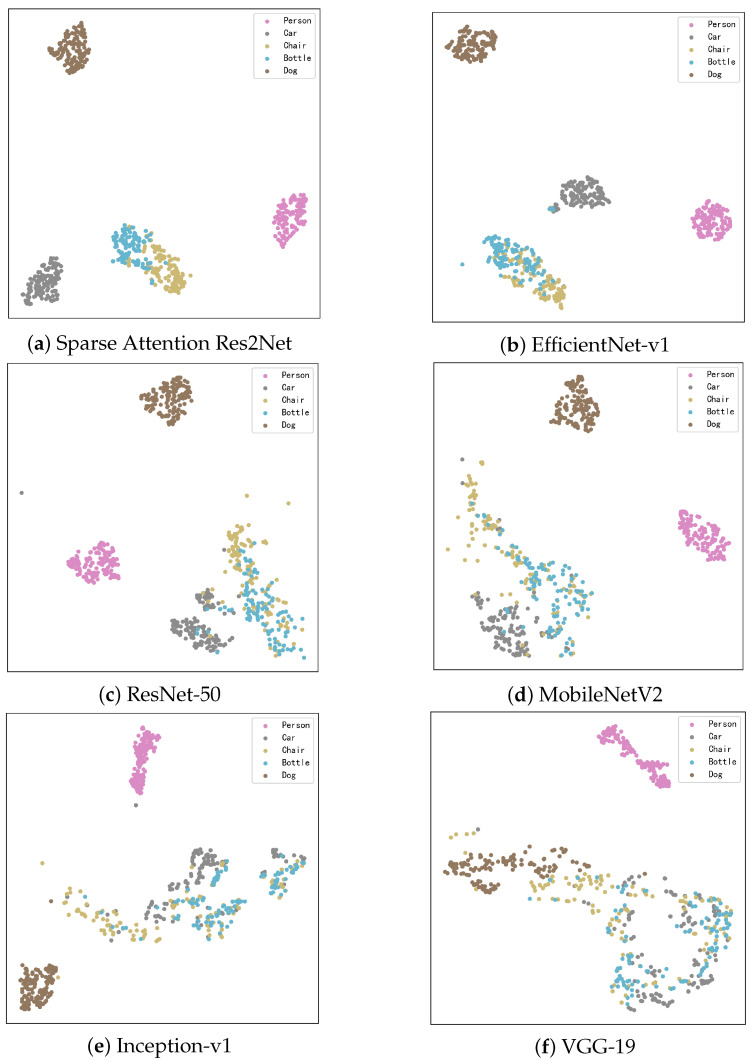
t-SNE visualization on comparative datasets.

Figure 9 presents the t-SNE visualization results of six models on the COCO dataset, offering an intuitive comparison of their feature space performance. The Sparse-Attention-driven Res2Net model exhibits compact clusters with relatively clear boundaries in the feature space, demonstrating strong class separation and fine-grained feature capture capabilities. While some overlap remains between different categories, the overall separation is significantly better than that of other models.

In comparison, EfficientNet-v1 and ResNet-50 perform moderately well in the t-SNE visualizations. They successfully distinguish most categories but exhibit noticeable overlap at the cluster boundaries, particularly between similar categories. This suggests that their feature extraction capabilities are limited in complex background conditions, affecting their ability to differentiate subtle differences.

Lightweight models MobileNetV2 and Inception-v1 show more dispersed cluster distributions with fuzzy boundaries and significant overlap between categories. This indicates weaker feature representation capabilities, making it difficult for these models to capture fine-grained features in complex backgrounds. These limitations are particularly pronounced in scenarios involving similar categories or substantial background interference.

VGG-19 performs the worst, with loose and heavily overlapping clusters in the t-SNE plot. Many category samples are almost entirely mixed, reflecting poor feature extraction and generalization capabilities. This model struggles to handle the complex and diverse scenes in the COCO dataset, resulting in inferior classification performance.

Overall, these t-SNE analyses highlight differences in cluster compactness and boundary clarity across models in the feature space. They provide further support, from the perspective of data distribution, for the conclusion that the Sparse-Attention-driven Res2Net achieves superior performance on the COCO dataset.

### 4.3. Ablation Experiment

Ablation experiments were designed to evaluate the individual contributions of key components in our proposed model. By systematically modifying or removing elements, we aimed to gain insights into their impact on performance and validate their necessity in achieving high accuracy and robustness. The results of the ablation experiment in this article are shown in Table 8.

The baseline model, Res2Net, was selected as a foundation due to its multi-scale feature extraction capabilities. The proposed method enhances Res2Net with Sparse Attention mechanisms and advanced multi-scale feature aggregation, resulting in a notable accuracy improvement to 98.46%. This demonstrates the effectiveness of integrating these features for fine-grained classification tasks.

When Res2Net was replaced with traditional residual blocks, a significant performance drop was observed, with accuracy falling to 94.87%. This indicates the critical role of Res2Net’s multi-scale design in capturing detailed features. Furthermore, removing both Sparse Attention and multi-scale aggregation reduced accuracy to 91.52%, underscoring the combined importance of these components. Sparse attention selectively focuses on relevant regions, while multi-scale aggregation ensures the capture of features across varying resolutions.

Additional experiments targeted specific components. Reducing the connection density in the Sparse Attention mechanism led to an accuracy drop to 97.18%, emphasizing the importance of balanced connectivity for effective feature interaction. Similarly, reducing the resolution in the multi-scale feature aggregation process decreased accuracy to 95.78%, highlighting the need for detailed feature aggregation to maintain classification precision.

These ablation studies validate the necessity of each component in the proposed method. They also provide theoretical support for design decisions, demonstrating how Sparse Attention and multi-scale aggregation synergistically contribute to superior performance. This systematic evaluation reinforces the model’s design and highlights its advantages over conventional architectures.

### 4.4. Applicability of the Method Dataset in This Article

The dynamic Sparse-Attention-driven Res2Net model excels in tasks with clear features and prominent textures, particularly in fine-grained classification scenarios. Its ability to extract multi-scale features combined with a dynamic attention mechanism enables precise capture of both local and global information, significantly enhancing classification performance. This makes the model highly adaptable and generalizable to datasets with distinct texture differences or well-defined category boundaries.

However, in datasets with complex backgrounds, the model may be affected by distracting features, resulting in dispersed attention distribution or redundant features, which limit its classification capabilities. The increased presence of interference in complex scenes exacerbates the difficulty of feature extraction. Additionally, in small-sample or sparse data scenarios, the dynamic Sparse Attention’s reliance on adequate sample support for weight learning may lead to unstable attention distribution, negatively impacting the model’s performance and stability.

To address these challenges, improving the model’s adaptability is crucial. One approach is to enhance the attention mechanism by integrating global and local attention or incorporating contrastive learning techniques to strengthen feature representation and reduce the influence of background interference. Additionally, adopting methods such as adversarial data augmentation, transfer learning, and generative adversarial networks can effectively improve the model’s generalization ability and stability in small-sample tasks.

Further optimization of dynamic Sparse Attention can be achieved by introducing background suppression mechanisms to dynamically evaluate and mitigate the impact of distracting background features. Regularization techniques can also be employed to refine Sparse Attention distributions, enhancing robustness in complex data scenarios. These improvements will provide both theoretical and practical support for achieving superior performance across a broader range of datasets and tasks.

### 4.5. Limitations and Challenges of the Method Described in This Article

Although the Sparse Attention-enhanced Res2Net framework achieves a remarkable balance between multi-scale feature modeling and computational efficiency, it has certain limitations. First, its generalization ability in other domains remains to be validated, as its sparsification strategy and feature modeling may require adjustment to accommodate different data characteristics. Second, while Sparse Attention reduces computational complexity, its real-time performance might still be constrained when handling ultra-high-resolution images or operating in resource-limited embedded systems. Furthermore, the model’s reliance on high-quality annotated data increases the cost of data acquisition in practical applications, and the adaptability of the sparsification strategy in complex scenarios requires further optimization. Finally, hardware implementation of the framework may face performance bottlenecks in sparse matrix operations, necessitating further refinement to suit specific hardware architectures. These limitations provide avenues for future improvements and contribute to a more comprehensive evaluation of the method’s practical applicability and potential.

To address the demands of multi-resolution discernibility and real-time UAV vision detection, the Sparse Attention-enhanced Res2Net model can be extended and optimized in several ways. Introducing cross-scale feature fusion modules could enhance its ability to detect targets at varying scales. Adjusting the sparsification strategy would make attention computations more efficient, further reducing computational complexity. Optimizing the model size through quantization and pruning, combined with embedded hardware acceleration technologies, could improve real-time inference performance. Moreover, adversarial data augmentation and adaptive weighting mechanisms tailored to the complex environments faced by UAVs could enhance the model’s robustness across different scenarios.

## 5. Conclusions

This paper proposes a Sparse-Attention-driven Res2Net approach aimed at addressing challenges in fine-grained classification tasks, particularly the trade-off between model complexity and inference efficiency as well as limited ability to extract subtle features. By integrating a Sparse Attention mechanism, the model dynamically focuses on task-critical regions, enhancing fine-grained feature representation. Coupled with multi-scale feature aggregation, it captures both local details and global context effectively. The lightweight network design significantly reduces parameter count and inference time, balancing computational cost and accuracy.

We conducted extensive experiments on two benchmark datasets: Fruits-360, containing nine categories of fruits with complex textures and diverse shapes, and COCO, a large-scale dataset with complex and varied objects. The proposed method achieved superior classification accuracy and robustness compared to baseline models on both datasets. For example, on Fruits-360, the Sparse-Attention Res2Net reached an accuracy of 98.46%, outperforming EfficientNet-v1 and ResNet-50. Visualizations using t-SNE and class activation mapping further illustrate the model’s ability to learn discriminative features and focus on key image regions. Ablation studies demonstrate the individual contributions of the Sparse Attention module and multi-scale feature extraction blocks.

These results confirm the proposed method’s effectiveness and generalization across datasets of different complexity. Future work will explore integrating Sparse Attention with dynamic feature learning strategies, expanding to more diverse datasets, optimizing architectures for even higher efficiency, and applying ensemble methods to further enhance accuracy and robustness in fine-grained classification tasks.

## Figures and Tables

**Table 1:** Distribution of sample categories in the dataset.

Category	Training Set	Validation Set	Testing Set	Total Sample Size	Label
Avocado	400	70	70	540	0
Papaya	400	70	70	540	1
Pitahaya Red	400	70	70	540	2
Huckleberry	400	70	70	540	3
Cactus Fruit	400	70	70	540	4
Passion Fruit	400	70	70	540	5
Lychee	400	70	70	540	6
Tangelo	400	70	70	540	7
Walnut	400	70	70	540	8

Table notes: The dataset is evenly distributed across categories, ensuring balanced representation for training, validation, and testing.

**Table 2:** Distribution of model structure parameters.

Layer Name	Input Shape	Output Shape	Operation	Parameters	Activation	FLOPs
Conv1 + BN + LeakyReLU	(B, 3, 224, 224)	(B, 32, 112, 112)	7 × 7 Conv, Stride 2	∼4.7k	Leaky ReLU, slope = 0.01	∼110M
MaxPool	(B, 32, 112, 112)	(B, 32, 56, 56)	3 × 3 Max Pool, Stride 2	0	None	∼10M
Res2Net Block 1	(B, 32, 56, 56)	(B, 64, 56, 56)	Multi-scale Conv	∼55k	Leaky ReLU, slope = 0.01	∼120M
Res2Net Block 2	(B, 64, 56, 56)	(B, 128, 56, 56)	Multi-scale Conv	∼220k	Leaky ReLU, slope = 0.01	∼240M
Res2Net Block 3	(B, 128, 56, 56)	(B, 256, 56, 56)	Multi-scale Conv	∼880k	Leaky ReLU, slope = 0.01	∼480M
Sparse Attention	(B, 256, 56, 56)	(B, 256, 56, 56)	Sparse Attention	∼66k	Leaky ReLU, slope = 0.01	∼90M
Adaptive AvgPool	(B, 256, 56, 56)	(B, 256, 1, 1)	Avg Pool	0	None	∼0.1M
Fully Connected	(B, 256)	(B, 9)	Linear	∼2.57k	None	∼0.05M

Table notes: FLOPs are approximations based on input batch size *B. Parameters* marked with “∼” denote approximate values.

**Table 3:** Detailed network layer configurations of comparison models.

Model Name	Specific Network Layer Configuration
VGG-19 [33]	16 convolutional layers with 3 × 3 kernels, each followed by ReLU activation; 5 max-pooling layers with 2 × 2 windows; 3 fully connected layers including one Softmax output layer.
ResNet-50 [34]	One 7 × 7 convolutional layer with stride 2, followed by a 3 × 3 max-pooling layer with stride 2; four residual stages containing 3, 4, 6, and 3 residual blocks, respectively; each block includes three convolutions (1 × 1, 3 × 3, and 1 × 1); final global average pooling and fully connected output layer.
MobileNetV2 [35]	One standard 3 × 3 convolutional layer with stride 2; 17 inverted residual blocks incorporating depthwise separable convolutions; each block consists of a pointwise convolution and a depthwise convolution; final global average pooling and fully connected output layer.
Inception-v1 [36]	Five Inception modules, each containing parallel branches of 1 × 1, 3 × 3, 5 × 5 convolutions, and a max-pooling layer; feature maps are merged and dimensionality reduced by pointwise convolution; final global average pooling and fully connected output layer.
EfficientNet-v1 [37]	One standard 3 × 3 convolutional layer with stride 2; multiple MBConv blocks, the number depending on network depth; employs a compound scaling strategy to adjust width, depth, and resolution; final global average pooling and fully connected output layer.

Table notes: References in brackets correspond to the original papers describing the respective models.

**Table 4:** Test results and evaluation metrics.

Method	Accuracy (%)	Precision (%)	Recall (%)	F1 Score (%)	Parameters (M)	Inference Time (ms/Sample)
Sparse Attention Res2Net	98.46 ± 0.12	98.50 ± 0.10	98.40 ± 0.11	98.45 ± 0.09	∼4.5	∼4 ± 0.2
EfficientNet-v1	98.12 ± 0.15	98.20 ± 0.14	98.00 ± 0.13	98.10 ± 0.12	∼5.3	∼6 ± 0.3
ResNet-50	97.89 ± 0.20	97.90 ± 0.18	97.85 ± 0.19	97.88 ± 0.17	∼25	∼5 ± 0.4
MobileNetV2	93.32 ± 0.25	93.40 ± 0.23	93.30 ± 0.24	93.35 ± 0.22	∼3.4	∼3 ± 0.1
Inception-v1	92.68 ± 0.30	92.70 ± 0.28	92.65 ± 0.29	92.68 ± 0.27	∼5.6	∼4.5 ± 0.3
VGG-19	86.73 ± 0.35	86.80 ± 0.33	86.70 ± 0.34	86.75 ± 0.32	∼143	∼8 ± 0.5

Table notes: Parameters are denoted in millions (M). Inference time is measured as milliseconds per sample. Standard deviations indicate result variability across multiple runs.

**Table 5:** Comparison of FLOPs between standard global attention and Sparse Attention.

Attention Mechanism Type	Number of Attention Layers	Scope of Operation	Attention Layer FLOPs	Reduction Ratio (Sparse vs. Global)
Standard Global Attention	3	Channels × Full Spatial Area	∼200M	—
Sparse Attention (This Study)	3	Channels × Local Spatial Windows	∼90M	Approximately 55%

Table notes: FLOPs are reported per attention layer in millions (M). The reduction ratio indicates the percentage decrease in computational complexity achieved by Sparse Attention compared to standard global attention.

**Table 6:** Comparative dataset.

Category	Total Samples	Training Set	Validation Set	Test Set	Label
Person	1000	700	150	150	0
Car	1000	700	150	150	1
Dog	1000	700	150	150	2
Chair	1000	700	150	150	3
Bottle	1000	700	150	150	4
Total	5000	3500	750	750	—

Table notes: The dataset is divided into training, validation, and test sets with equal proportions across all categories. Labels represent class indices for classification tasks.

**Table 7:** Evaluation metrics of different models on the COCO dataset.

Model	Accuracy	Precision	Recall	F1 Score
Sparse Attention Res2Net	97.85% ± 0.12	97.89% ± 0.11	97.82% ± 0.13	97.86% ± 0.10
EfficientNet-v1	97.63% ± 0.15	97.68% ± 0.14	97.58% ± 0.16	97.63% ± 0.12
ResNet-50	97.21% ± 0.18	97.25% ± 0.17	97.18% ± 0.19	97.21% ± 0.15
MobileNetV2	94.87% ± 0.20	94.92% ± 0.18	94.83% ± 0.22	94.87% ± 0.17
Inception-v1	94.35% ± 0.25	94.39% ± 0.23	94.31% ± 0.26	94.35% ± 0.22
VGG-19	89.52% ± 0.30	89.57% ± 0.28	89.48% ± 0.32	89.52% ± 0.25

Table notes: Metrics are presented as mean ± standard deviation across multiple runs.

**Table 8:** Results of ablation experiment analysis.

Model	Accuracy (%)	Precision (%)	Recall (%)	F1 Score (%)	Parameters (M)	Inference Time (ms/Sample)
Proposed Method	98.46	98.50	98.40	98.45	∼4.5	4
Res2Net	96.32	96.40	96.20	96.30	∼4.2	3.8
Replacing Res2Net with Residual Blocks	94.87	94.90	94.80	94.85	∼3.9	3.5
Removing Sparse Attention and Multi-Scale Features	91.52	91.60	91.50	91.55	∼3.5	3.2
Reducing Sparse Attention Connection Density	97.18	97.20	97.10	97.15	∼4.3	4.1
Reducing Resolution in Multi-Scale Feature Aggregation	95.78	95.80	95.70	95.75	∼4.0	3.7

Table notes: Parameters are denoted in millions (M). Inference time is measured as milliseconds per sample.

## Data Availability

The data presented in this study are available in the article.
